# Cutting a Drop of Water Pinned by Wire Loops Using a Superhydrophobic Surface and Knife

**DOI:** 10.1371/journal.pone.0045893

**Published:** 2012-09-24

**Authors:** Ryan Yanashima, Antonio A. García, James Aldridge, Noah Weiss, Mark A. Hayes, James H. Andrews

**Affiliations:** 1 Department of Chemistry & Biochemistry, Arizona State University, Tempe, Arizona, United States of America; 2 School of Biological and Health Systems Engineering, Arizona State University, Tempe, Arizona, United States of America; 3 Department of Physics and Astronomy, Youngstown State University, Youngstown, Ohio, United States of America; Queen’s University at Kingston, Canada

## Abstract

A water drop on a superhydrophobic surface that is pinned by wire loops can be reproducibly cut without formation of satellite droplets. Drops placed on low-density polyethylene surfaces and Teflon-coated glass slides were cut with superhydrophobic knives of low-density polyethylene and treated copper or zinc sheets, respectively. Distortion of drop shape by the superhydrophobic knife enables a clean break. The driving force for droplet formation arises from the lower surface free energy for two separate drops, and it is modeled as a 2-D system. An estimate of the free energy change serves to guide when droplets will form based on the variation of drop volume, loop spacing and knife depth. Combining the cutting process with an electrofocusing driving force could enable a reproducible biomolecular separation without troubling satellite drop formation.

## Introduction

In the past few years there has been a spectacular growth in the number of scientific articles describing the manufacture of water repellent surfaces, also known as superhydrophobic surfaces, for a wide range of consumer and industrial uses [1]–[3]. Similarly, droplet microfluidics, which is generally described as the creation and manipulation of defined droplets in an insoluble continuous phase, is a popular topic in biotechnology, analytical instrumentation, high-throughput screening, and other instrumentation development. A particular focus is using the unique surfaces to enable the manipulation of individual drops so that a complex mixture can be rapidly and inexpensively resolved into individual components. In general, a major challenge in biomolecular separations is to separate a large number of key proteins from biological fluids for a variety of clinical and biotechnological applications.

Multiprotein separation is vital for the detection of important proteins that provide valuable information on gene expression and can serve as early signals of a disease state. Currently, technological solutions are limited to using specific labels (*e.g.*, ligands or antibodies) or an array of instruments with accompanying sample preparation steps, which usually require expert handling. Finding a rapid, efficient and simple means of separating components in a small sample, such as a drop, without using channels, stationary phase, gels or other transfer media is a two-pronged problem. One needs, firstly, a suitable means of generating conditions within the drop for separating molecules and, secondly, a means of collecting one or more components separated from the rest. Previously, the generation of a pH gradient suitable to create isoelectric focusing in a drop sitting on a superhydrophobic surface was demonstrated [4]. Although a micropipette system or some other suitable, additional instrument could remove the isolated protein, our group became interested in developing a strategy to divide the drop without generating undesired mixing effects or satellite drops. Simply stretching the drop in order to divide it generates a meniscus shape in the liquid leading to a thinning of the bridge followed by a catastrophic rupture often resulting in small satellite drops as can be seen in liquid jets [5]–[9]. Instead, a means to cut the drop at a particular location with a superhydrophobic knife would be more helpful in achieving the desired goal of separating molecular components within a single drop. Satellite droplets are often formed when the Weber number (*We  =  ρU^2^L/γ*) of a system is greater than 1. As a comparison, Weber numbers for our system are on the order of 1×10^−5^. This is mostly due to the very slow speed of the knife descending upon the stretched droplet. Bormashenko & Bormashenko [10] have recently demonstrated that for an unpinned droplet, the speed of a superhydrophobic knife is important when cutting a coated liquid marble or a liquid drop on a superhydrophobic surface. However, our parallel and independent research effort is focused on drop cutting when the system is at equilibrium and while the drop is pinned by two wire loops that can function as electrodes. The physics of this drop cutting method is rather simple compared to other droplet formation and manipulation strategies (flowing streams–Rayleigh-Tomotika analysis [11]–[13] and electrowetting–fully assessed in 2003 [14]). It depends entirely on the difference in the surface free energy between a distorted drop shape and two separate drops pinned to the wire loops. Our method does not rely on standard flow models or the viscosity of the two fluids of the liquid/air interface. Furthermore, our droplets are not being stretched enough such that they can begin to behave like an unstable fluid filament. In this strategy, a force applied to the surface using a slow-moving superhydrophobic knife–after components are spatially separated using isoelectric focusing or some other separation method–assists the drop-cutting step and leads to the creation of two drops that can be further processed or collected and analyzed.

**Figure 1:**
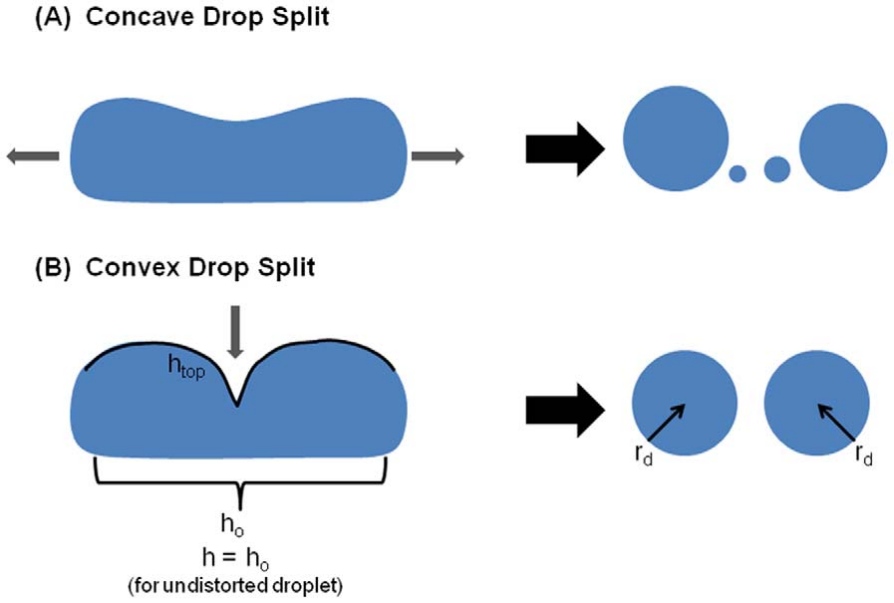
Schematic contrasting splitting a drop by pulling each end with the method of cutting with a superhydrophobic knife. Both drops lie on superhydrophobic surfaces. A simple rectangle can estimate the shape and contour of the drop (*h_top_* for the top contour of the stretched drop, and *h_o_* for the bottom contour of the stretched drop). The split drop results in two equally sized spheres, both of radius *r_d_* (equal to the radius of the wire loops used to pin and stretch the original droplet).

## Materials and Methods

### Superhydrophobic Surfaces

Polyethylene was used for the cutting experiments using superhydrophobic polyethylene knives. For the experiments with zinc superhydrophobic knives, superhydrophobic Teflon slides (Electron Microscopy Science, Hatfield, PA) were used. The Teflon slides proved to be more resilient and smoother surfaces than the polyethylene. The contact angles for these Teflon slides were found to be about 135°, typical of Teflon.

**Figure 2:**
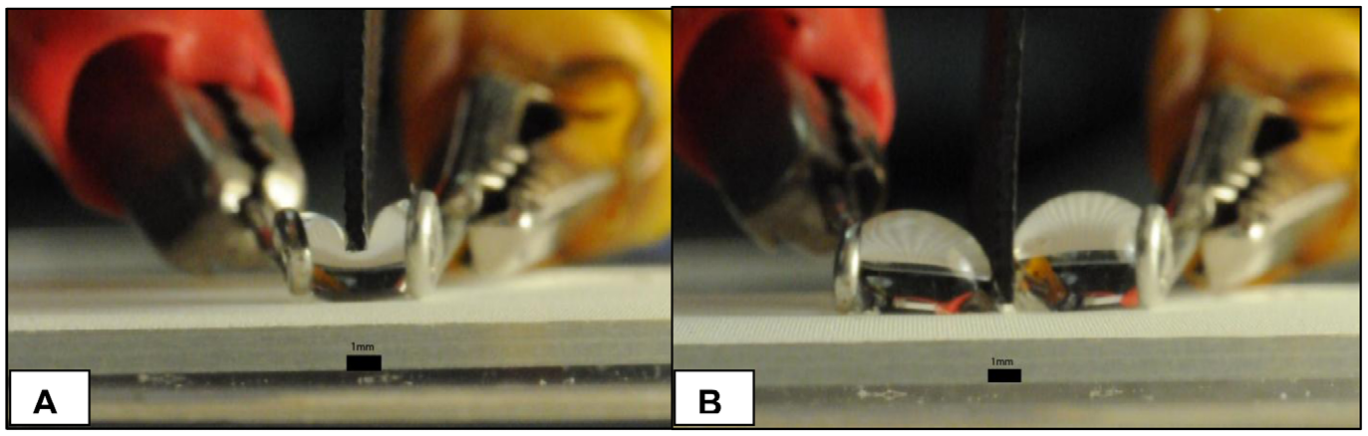
Images from video footage of drop cutting experiments using a superhydrophobic knife on superhydrophobic surfaces: (A) 15 µL water drop on a Teflon glass surface separated 3 mm by wire loops is not cut into two drops and bounces back after the zinc superhydrophobic knife is elevated, (B) 50 µL drop separated by wire loops by 8 mm does cut into two drops. Scale bar is 1 mm in both images.

### Superhydrophobic Knives

Polyethylene and zinc were used as starting materials to make superhydrophobic knives. Polyethylene knives were made superhydrophobic using a solvent casting method as described previously [15]. The metallic superhydrophobic blades were prepared using an electroless galvanic deposition procedure [16]. Copper and zinc sheets 0.020 inches thick were cut into small blades, cleaned with acetone, ethanol, DI water and then air-dried with nitrogen. The metal blades were dipped in a 10 mM aqueous solution of silver nitrate for approximately 20 seconds, then washed with water and air-dried. The blades were then dipped into a 1 mM solution of HDFT (3,3,4,4,5,5,6,6,7,7,8,8,9,9,10,10,10-heptadecafluoro-1-decanethiol) in dichloromethane for approximately five minutes. They were then rinsed with dichloromethane and air-dried. This fabrication method for producing the superhydrophobic blades has been shown previously to have contact angles of about 173° [16]. The drop splitting mechanism had been demonstrated previously [4], whereby a drop of DI water was held stationary by two circular wire hoops with nominal diameters of 2 mm and wire thicknesses of 0.66 ± 0.02 mm. The drop was stretched lengthwise along the superhydrophobic surface by increasing the distance between the two wire hoops. Distances varied between 4.5 mm and 12 mm, while the volume of the drop varied between 15 and 70 µL. The typical speed of the descending superhydrophobic knife was about 0.35 mm/sec. Extreme close-up images of the droplet splitting were captured on video using a Nikon Digital SLR D5000 camera with an AF Micro Nikkor 105 mm 1∶2.8D lens. The video was taken at 24 fps, at a resolution of 1280×720. Images were analyzed using ImageJ software (Version 1.40, National Institutes of Health, Bethesda, MD). Calibrations of the length scale in the images were based on known sizes of the wire loops used to hold the water drops on either end. Surface profile lengths were obtained using built-in, freeform line drawing and measurement tools.

**Figure 3:**
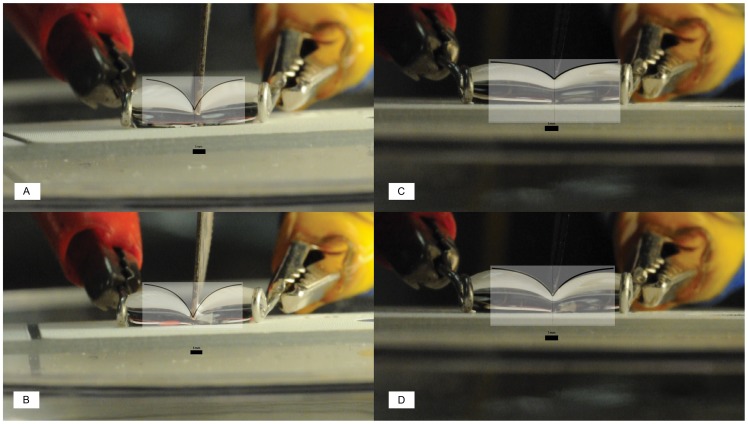
Images (A-D) show a curve drawn using Equation (5) superimposed on top of an example still from a video. The curves and droplet profiles correspond closely. All scale bars are 1 mm. (**A**) A droplet with a volume (*V_s_*) of 60 µL and with separation distance (*h_o_*) of 8.5 mm. The normalized time parameter, *B,* from Equation (5) is set to 0.70 for this curve. The parameters *A* and *C* from Equation (5) are both set to 1 in all images for simplicity. (**B**) *V_s_* = 60 µL, *h_o_* = 10.5 mm, and *B* = 0.75 (**C**) *V_s_* = 60 µL, *h_o_* = 10.0 mm, and *B* = 0.30 (**D**) *V_s_* = 60 µL, *h_o_* = 10.0 mm, and *B* = 0.40. Images (**C**) and (**D**) are of the same droplet.

## Results and Discussion

Understanding the closely related area of liquid jet breakage through experiments and theory development continues to be a relatively active research topic, but there is much less work involving splitting a single drop. Liquid jet breakup research does show that unless vibrations are carefully controlled [6], [8], satellite drops are formed. Stretching a drop (Figure 1 – top) cannot be controlled unless an additional force, such as through the use of controlled vibrations, is superimposed. High throughput flowing droplet formation microfluidic research also shows that careful control of focusing conditions is important in order to control the neck between droplets and hence to avoid satellite drops [17], [18]. Satellite formation in microfluidics is similar to what occurs in free liquid jets, and it can be generally attributed to the action of droplets coming apart while liquid is held in a thin bridge between the two larger droplets.

**Table 1:** Upper Limit Predictions and Results.

Volume of Drop *V_s_* (µL)	Measured Separation Distance*h_o_* (mm)	Upper Limit (mm)	Measured *h_top_* value (mm)	Did Drop Split in Next Frame?
50	8.3	12.9	11.0	No
50	8.3	12.9	12.9	***Yes***
60	9.3	14.2	14.2	No
60	9.2	14.2	14.2	No
60	9.2	14.2	14.3	No
60	9.3	14.2	14.3	***Yes***
60	10.7	15.0	12.8	No
60	10.7	15.0	12.9	***Yes***
70	13.0	17.1	14.5	No
70	13.1	17.1	14.6	***Yes***

Examples of upper limit predictions as compared to ImageJ measured values and observed drop cutting for a zinc coated superhydrophobic knife and a Teflon superhydrophobic surface (based on the 2-D model). Out of 15 videos capturing drop splitting, there were 74 photos analyzed. In 17 of them (23%), the measured values for h_top_ exceed the calculated upper limit, however all of those were still within 7% of the calculated upper limit.

As shown in Figure 1 (a), some information can be gleaned from droplet formation via liquid jet as it generates a concave meniscus that can create satellite drops due to the elastic response of the drop during breakage, which is characterized by oscillations [6], [8]. In distinct contrast of spontaneously generating a concave meniscus, our technique involves pressing down on the liquid cylinder with a superhydrophobic knife to form two convex menisci, as shown in Figure 1 (b). This form of cutting does not create satellite drops since the surface tension driving force “folds” the liquid inwards on each daughter drop respectively [16].

When slowly cutting a drop pinned on each side with a superhydrophobic knife, the slicing or cutting action gradually eliminates the bridge while allowing time for the liquid to be folded into one droplet or the other. For a given separation distance between the wire loops, the superhydrophobic knife can be slowly lowered until it touches the superhydrophobic surface and then slowly raised back above the drop(s) surface, indicating a reversible system until breakage–in contrast to a liquid jet. A droplet can either split, if shape distortion is sufficiently achieved by the blade, or the drop can resume its original shape upon blade removal.

Figure 2 (a) shows a typical still image from digital video for a drop that could not be cut, while Figure 2 (b) shows a drop at the point of being cut in two. Still sequences from videos were collected to document the distortion of the upper surface by the superhydrophobic knife in order to compare the profile to an appropriately simple2-D thermodynamic model prediction of the range of conditions that would favor cutting of the drop.

The wire loop separation favorable to drop splitting and the contour lengths of the droplets are of particular interest. The following analysis creates a framework to guide drop cutting and provides a range of values needed to achieve cutting.

From the analysis of Young and the mathematics of Laplace, the change of the shape of a drop of water is determined by balancing pressure and surface tension in order to achieve a fluid static condition. For our analysis, we only examine two different shapes – a sphere and a cylinder. Assuming a perfectly superhydrophobic surface and that a drop elongated by connecting to two wire hoops creates a cylinder, the energy for both static states at constant volume can be described.

Because no material is lost in the drop splitting process, the volume, *V*, remains constant throughout, but can be expressed for clarity by: *V*_s_ = 4/3 *πr_s_* ^3^ for the original sphere, *V_d_* = 1/2*V*_s_ = 2/3 *π_d_* ^3^ for the daughter droplets, (assuming equal splitting, subscript *d* for daughter droplets, of which there are two) and *V_c_* = *πr_c_* ^2^ *h* for the cylindrically stretched droplet, (subscript *c* for cylinder) where *h* is the length of a stretched droplet. See Figure 1 and the discussion below.

Surface area varies for the different configurations and gives rise to significant differences in the Gibbs Free Energy, which provides a framework or waypoints to understand the cutting process. Given the surface area of the original sphere, *A_s_* = 4*πr_s_* ^2^, the Gibbs Free Energy to form the spherical interface is then, Δ*G* = 4*πr_s_* ^2^ *γ*. Similarly, the surface area for a cylinder, *A_c_* = 2*πr_c_h* + 2*πr_c_* ^2^, results in Δ*G* = (2*πr_c_h* + 2*πr_c_* ^2^)*γ*. For the process of stretching a droplet in preparation for splitting, the droplet shape changes from a sphere to a cylinder, which is a positive free energy change, Δ*G* = 2*πγ*(*r_c_h* + *r_c_* ^2^–*r_s_* ^2^). Setting the volumes equal to each other, solving for *r_s_* and substituting gives us the following relation:
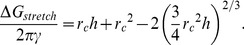
(1)


For the process of cutting the cylinder into two equal size spheres, the energy change is calculated by comparing the energy of a cylinder with two spheres equal to the original volume defined above:
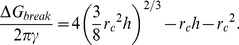
(2)


This free energy change can be positive or negative depending upon the ratio of the cylinder radius to its height (*r_c_*/*h*). Assuming that the cylinder starts at a relatively small value of *h* with respect to *r_c_*, by stretching the cylinder so that *h* is 4–5 times the radius, the free energy change for this process is negative meaning that the drop would minimize its free energy by forming two separate drops. This relatively simple two stage model fully and simply describes the 3-D energetics of the system.

To provide a quantitative assessment for the imaging data of the drop cutting, a reduced model is needed since full 3-D imaging is not desirable or especially feasible. The important outcome is to design a rubric to identify conditions favoring drop cutting that can be deduced from the 2-D free energy analysis and can be effectively compared to experiments by imaging of a side view of the cutting process. In this model, the energy change considers only the circumference of the cylinder and spheres that are at the liquid/air interface, and assumes that the surface tension force acts across the length of the interfacial line. Here, the liquid/air cylinder circumference is considered to be only the top (*h_top_*) and bottom (*h_o_*) lengths (see Figure 1). For this 2-D analysis, the liquid/air circumference prior to splitting is a rectangle, and if the drop splits, the liquid/air circumference of the two drops is two half-circles. The ends of the rectangle and other half of the circles pinned by the wire loops during the splitting process are not considered in this model since these surfaces undergo no changes in interfacial energy. The surface length of *h_top_* is the only length assumed to be increasing due to the action of the superhydrophobic knife. The 2-D model yields the following free energy equation assuming that the drop splits evenly:
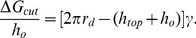
(3)


Thus, for a given length of a cylindrically shaped drop, the creation of a higher interfacial length (*h_top_*) can lead to a negative free energy change. As the drop is stretched more due to a wider spacing between the wire loops, it is comparatively easier to slice the drop with the superhydrophobic knife since the increase in the needed interface length as a percentage of the original length is decreased. The lower limit to achieve drop splitting is found by setting equation (3) equal to zero and solving for *h_top_* in terms of *V*. The minimum value of *h_top_* in order to achieve drop cutting is:
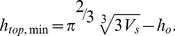
(4)


However, this underestimates the value of *h_top_* needed when the drop volume is larger. The upper limit in principle is the maximum distortion the knife can impose upon the droplet. To calculate the upper limit, an arc length must be calculated for the top contour of the drop. For the upper limit estimate, the following general equation is used to approximate the drop profile upon cutting near the knife:
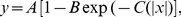
(5)where *A* corresponds to the height of the droplet’s upper surface above the Teflon slide, which can be obtained from the diameter of the hoops, *d*; *B* corresponds to normalized time assuming the knife is moving at a constant velocity, and is equal to the value 1 for the purposes of calculating the upper limit; and *C* is the ratio *h_o_*/*V_s_*. Figure 3 shows four typical still images from the videos with a curve drawn from equation (5) superimposed to demonstrate how this equation reasonably describes the droplet profile during splitting. The upper limit of h_top_ is found from the droplet being distorted to the maximum by the superhydrophobic knife. Taking the arc length of the drop profile at the point when the knife has descended as far as it can before touching the Teflon slide surface, and substituting in the meaningful variables for *A*, *B*, and *C* as described above gives:
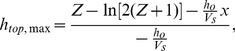
(6)where:



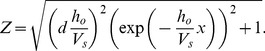
(7)
Table 1 compares calculated upper limits for various droplet scenarios with the measured values of *h_top_* using the ImageJ software and shows whether the deformed drop was about to split in the next frame of the video.

For a range of separation distances of 3–13 mm and drop sizes of 15–70 µL, the measured *h_top_* values when drop cutting occurs more closely follow the upper limit prediction. Our observations also show that measured *h_top_* values which do not well exceed the lower limit are insufficient to generate drop splitting. Larger droplets can achieve splitting before reaching the upper limit due to their instability. We recommend that a separation distance between wire loops or other pinning surfaces of 4–5 times the original drop radius is a good rule of thumb to cut drops in this fashion, which is similar to the geometric rule of thumb as noted for laminar jets [8], [9].

### Conclusions

Using a variety of techniques to fabricate superhydrophobic surfaces and knives, a drop of water pinned to wire loops could be cut in a gentle fashion so that no satellite drops are formed. This technique complements a previous publication [4] describing how isoelectric focusing can be conducted on an aqueous drop resting on a superhydrophobic surface. Drop cutting is a further step that completes the separation of proteins that preferentially migrate to an electrode during focusing. A 3-D model is presented and experimental data compared to a 2-D thermodynamic model which can guide the separation distance of the wire loop electrodes needed for cutting as a function of the initial drop volume. Surfaces that repel other types of liquids, such as superoleophobic surfaces, could also cut organic or ionic liquids via the method described.
